# Clinical Epidemiological Characteristics and Risk Factors for Severe Bronchiolitis Caused by Respiratory Syncytial Virus in Vietnamese Children

**DOI:** 10.1155/2021/9704666

**Published:** 2021-11-15

**Authors:** Sang Ngoc Nguyen, Thuy Ngoc Thi Nguyen, Lam Tung Vu, Thap Duc Nguyen

**Affiliations:** Haiphong University of Medicine and Pharmacy, Haiphong, Vietnam

## Abstract

**Introduction:**

Bronchiolitis is the most prevalent cause of hospitalization in infants under the age of 12 months. The disease is caused by respiratory syncytial virus (RSV) infection, which can cause breathing difficulties and respiratory failure. Therefore, it is necessary to discover the risk factors of severe bronchiolitis to diagnose and treat promptly. This study is aimed at describing the epidemiological characteristics and clinical features of acute bronchiolitis caused by RSV and assessing the related factors to severe acute bronchiolitis in studied patients.

**Methods:**

A descriptive cross-sectional study was carried out in Haiphong Children's Hospital, Haiphong, Vietnam, for one year, from October 1, 2016, to September 30, 2017. All bronchiolitis admissions < 2 years were included.

**Results:**

377 children were evaluated, including 261 boys and 116 girls; children under 6 months accounted for the highest proportion (57%), and 47 (12.5%) of all patients had severe disease. Wheezing was the main reason to be taken to the hospital 261 (69.2%). Clinical symptoms of acute bronchiolitis such as cough, tachypnea, and runny nose were found in all patients. Bronchiolitis cases increased in the winter-spring season, and the highest registered number of patients was 42 in March. Image of bronchiolitis on chest X-ray was found in all patients, and air trapping lung was found in 124 (32.9%) patients. The risk factors included age (≤6 months), low birth weight, preterm birth, nonbreastfeeding for the first six months, early weaning, and exposition to cigarette smoke increased the severe disease (*p* < 0.05).

**Conclusion:**

The number of hospitalized infants with bronchiolitis caused by RSV has an upward trend during the winter-spring season (from October to March). This study confirms that age, preterm birth, breastfeeding under 6 months, history of exposure to cigarette smoking, low birth weight, having sibling(s) under five years old going to kindergarten, history of undergoing cesarean section, history of mechanical ventilation, poor living condition, and maternal education are 10 risk factors of severe bronchiolitis caused by RSV.

## 1. Introduction

Bronchiolitis is the most prevalent cause of hospitalization in infants under the age of 12 months, resulting in a significant increase in expenditure to healthcare systems and families [1, 2]. Acute bronchiolitis is the inflammation of the bronchioles, which is most typically caused by a virus and is common in children under the age of two [3–5]. The disease is distinguished by its regularity of incidence, which is highest during the winter months of November to March [6]. Bronchiolitis is characterized clinically by expiratory breathing difficulties in babies, while cough, tachypnea, hyperinflation, chest retraction, broad crackles, and wheezing are several untypical symptoms. The disease has a diverse and complex clinical course, whose symptoms can range from mild to severe and quickly lead to respiratory failure [7]. Therefore, pediatric patients need to be diagnosed early and treated promptly to avoid mortality.

Bronchiolitis is mainly caused by a viral infection, especially respiratory syncytial virus (RSV) [8–11]. RSV is a negative-sense, single-stranded RNA virus belonging to the Pneumovirus genus in the *Paramyxoviridae* family with two antigenically distinct A and B subtypes, which is based on the reactivity of the F and G surface proteins to monoclonal antibodies [12–14], and can cause several symptoms, ranging from moderate upper respiratory tract infections to severe and possibly life-threatening lower respiratory tract infections, which may necessitate admission and mechanical ventilation. Bronchiolitis caused by RSV infection can manifest itself in a number of ways, ranging from moderate upper respiratory tract infections to severe and possibly life-threatening lower respiratory tract infections requiring hospitalization and mechanical ventilation [15].

By the age of two, the majority of children will have had at least one RSV infection [11]. While most acute bronchiolitis cases are mild and can be cured by home treatment [16, 17], acute respiratory distress syndrome causes 2-3% of pediatric patients to be admitted to hospitals, with 5% of them requiring transfer to intensive care units. The death rate of severe bronchiolitis is 1-7% and up to 30-40% in children at risk of preterm birth, bronchopulmonary dysplasia, and congenital heart defects. Many influencing factors lead to severe bronchiolitis (such as crowded living environment, inhalation of cigarette smoke, the lack of breastfeeding, and congenital heart defects) with longer hospitalization time and higher death rate [18, 19].

Knowledge of epidemiological data, the patient's age, clinical examination, and insight into the disease's risk factors are frequently sufficient aspects to identify severe levels of bronchiolitis as a clinical syndrome in everyday practice.

To that end, we propose the epidemiological characteristics and clinical features of acute bronchiolitis caused by RSV in pediatric patients at Haiphong Children Hospital from October 1, 2016, to September 30, 2017. Moreover, the current study is also aimed at assessing the related factors to severe acute bronchiolitis in studied patients.

## 2. Materials-Methods

A descriptive cross-sectional study was carried out for a one-year period, from October 1, 2016, to September 30, 2017. The study site was Haiphong Children's Hospital in Haiphong City, Vietnam.

### 2.1. Study Population

Charts of 377 patients diagnosed with bronchiolitis caused by respiratory syncytial virus infection were reviewed.

### 2.2. Inclusion Criteria

The study enrolled pediatric patients who suffered from bronchiolitis caused by respiratory syncytial virus infection and were treated in Haiphong Children's Hospital according to diagnostic criteria of the American Academy of Pediatrics (AAP) in 2014 [20], as follows:

- Inflammation of the upper respiratory tract: fever, cough, sneezing, and rhinorrhea
- Progressing to tachypnea, chest indentation, or intercostal muscle pull within 48 to 72 hours. Signs of air stasis were probably seen in clinical examination or chest X-ray
- Wheezing for the first or second time
- Lung examination: hissing, rhonchi, or crackles were heard mainly in the exhalation. There were possibilities of decreased vesicular murmur or no rale heard

Diagnosis the severity of bronchiolitis is based on the Modified Cincinnati Bronchiolitis Score (MCBS).  Table 1 lists the specifics of signs/symptoms for the diagnosis of bronchiolitis.

### 2.3. Exclusion Criteria

Children were excluded if they had wheezing more than two times or had a diagnosis of bronchial asthma. Additionally, if the child's age did not match with the ages included in this research, or there was no agreement of the family on participating in the research.

### 2.4. Methodology

On admission, all children had standardized sample collection. This includes using a nasopharyngeal swab to isolate viruses. A consistent methodology was used to collect and evaluate all samples: The RSV was detected in the nasopharyngeal swab samples collected at the time of admission using rapid direct immunofluorescence.

### 2.5. Data Analysis

The data were analyzed by Statistical Package for Social Sciences (SPSS) software version 26.0. To analyze the association between categorical variables, Pearson's chi-square test was used. All data are presented as mean ± standard error of the mean.

### 2.6. Ethical Approval

Approval for the study was obtained from the Medical Ethics Council of Haiphong University of Medicine and Pharmacy, and informed consent was obtained according to the Declaration of Helsinki.

## 3. Results

There were 377 children from 1 to 24 months old admitted to Haiphong Children's Hospital, Haiphong, Vietnam, over the one-year period from October 1, 2016, to September 30, 2017.  Table 2 illustrates the epidemiological features of all studied patients. Out of the total number of patients, 261 (69.2%) were males, and 116 (30.8%) were females. All children evaluated during the research are divided into four groups based on the age distribution of bronchiolitis incidence. Children under the age of 6 months made up the first group, children aged 6 to under 12 months made up the second group, children aged 12 to under 18 months made up the third group, and children aged 18 to 24 months made up the fourth group. In our study, there were 215 (57%) children in the first group, 84 (22.3%) children in the second group, 52 (13.8%) children in the third group, and 26 (6.9%) children in the fourth groups. Regarding the severity of bronchiolitis, 330 (87.5%) patients were suffering from mild-moderate bronchiolitis, and 47 (12.5%) were infants with severe bronchiolitis.  Table 3 indicates the significant differences in acute bronchiolitis severity across four age groups (*p* < 0.01).

**Figure 1:**
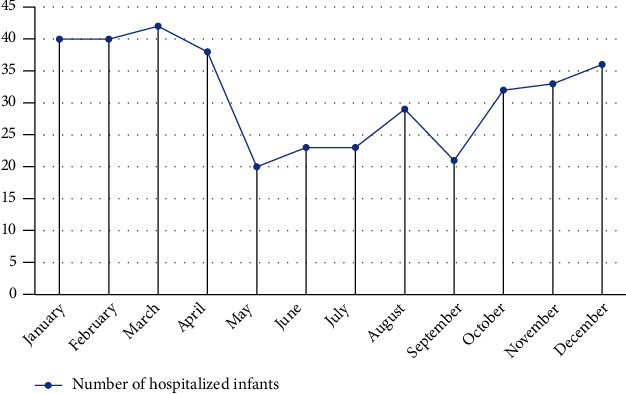
Monthly distribution of bronchiolitis patients in a one-year period.

In our study, patients with bronchiolitis were admitted at any time of year, although the highest number of patients admitted was in March, with 42 (11.1%) admitted, followed by January and February, with 40 (10.6%) admitted. In addition, only 20 individuals were admitted to the hospital in May owing to bronchiolitis. Thus, Figure 1 depicts the monthly distribution of bronchiolitis patients over the research period.  Table 4 shows that wheezing was the most frequent symptom when patients were hospitalized, accounting for 261 (69.2%) of all patients, whereas wheezing and fever were the least common, accounting for 11 (2.9%) of all patients. Clinical symptoms and signs such as wheezing, labored breathing, tachycardia, and sibilant rales were present in virtually every pediatric patient with acute bronchiolitis, especially tachypnea, runny nose, and cough was seen in 100% of patients. In contrast, just 3 (0.8%) of the patients exhibited gray skin, and 50 (13.3%) had sonorous rales (see Table 5). All findings indicated bronchiolitis in terms of chest X-ray images, while 124 (32.9%) cases presented air trapping lungs (see Table 6).

In children with bronchiolitis, we investigated a variety of potential risk factors related to severe bronchiolitis (Table 7). The following risk factors were independently associated with severe bronchiolitis, including age, preterm birth, breastfeeding under six months, history of exposure to cigarette smoking, low birth weight, having sibling(s) under five years old going to kindergarten, history of undergoing cesarean section, history of mechanical ventilation, poor living condition, and maternal education. Only sex exhibited no relationship with severe bronchiolitis among all variables investigated (*p* > 0.05).

## 4. Discussion

Virus infections are the leading cause of bronchiolitis in children worldwide, and RSV is well recognized as the most common virus in acute bronchiolitis in infants hospitalized [21]. In our study, boys accounted for 69.2%, which was consistent with the research of Ghazaly and Nadel [22], at 63%. Children under the age of 12 months were the most common age group of patients hospitalized with bronchiolitis, with children under the age of 6 months being the most prevalent, at 57%. We noted that there were 12.5% of cases of acute bronchiolitis presented as severe bronchiolitis.

Looking at the monthly distribution of infants hospitalized due to acute bronchiolitis in a one-year period, overall, there was a common increasing tendency over the winter-spring season (from October to March). The highest figure of infants admitted was 42 patients in March, followed by 40 patients in January and February. The number of hospitalized infants has seen a remarkable decline before hitting the lowest point in May, with 20 cases. Our findings indicated that the number of infants admitted with acute bronchiolitis significantly increases in the winter-spring time, while it gradually decreases in the rest of the year. Similarly, Bakalovic et al. [6] discovered that the winter season had the highest number of cases. In particular, the number of patients with bronchiolitis started to increase on November 15 (9.7%), and the number of patients gradually decreased after February, with the highest figure in January, at 29 (18.7%).

To determine the risk factors for severe bronchiolitis caused by RSV, we examined 11 variables between children with severe acute bronchiolitis and those with mild to moderate acute bronchiolitis, including sex, age, preterm birth, breastfeeding under six months, history of exposure to cigarette smoking, low birth weight, having sibling(s) under five years old going to kindergarten, history of undergoing cesarean section, history of mechanical ventilation, poor living condition, and maternal education. Table 7 shows the analysis of the risk factors independently linked with severe bronchiolitis in children aged two years or less. We noted that all variables except sex were risk factors associated with severe bronchiolitis. Especially, 4 factors were found to have strong relationship with severe bronchiolitis, including low birth weight (OR = 13.3; 95% CI = 4.15-42.77; *p* < 0.01), history of mechanical ventilation (OR = 10.14; 95% CI = 2.19-46.8; *p* < 0.01), poor living condition (OR = 9.4; 95% CI = 3.03-29.5; *p* < 0.01), and the history of exposure to cigarette smoking (OR = 7.4; 95% CI = 1.45-37.96; *p* < 0.01). Nicolai et al. also indicated that preterm newborns aged less than three months, low birth weight, and maternal smoking were the factors that increase the risk of severe bronchiolitis [21]. Moreover, Robledo-Aceves et al. [19] noted that exposure to cigarette smoking was associated with severe bronchiolitis (OR = 3.5; 95% CI = 1.99-6.18; *p* = 0.0001). Their study also demonstrated that maternal cigarette smoking and living in overcrowded conditions also increase the risk of infants admitted with severe bronchiolitis. With the same conclusion, Leem et al. also indicated that low socioeconomic status was one of the risk factors of severe bronchiolitis [23]. However, our findings showed that only gender was not associated with severe bronchiolitis (*p* > 0.05), while several studies indicated that gender was associated with severe bronchiolitis [19, 21].

The main limitation of this research is that the local characteristics of our study, as well as the diversity of children represented, are limiting factors in drawing definite and generalizable conclusions.

## 5. Conclusions

The number of hospitalized infants with bronchiolitis caused by RSV has an upward trend during the winter-spring season (from October to March).

This study confirms that age, preterm birth, breastfeeding under six months, history of exposure to cigarette smoking, low birth weight, having sibling(s) under five years old going to kindergarten, history of undergoing cesarean section, history of mechanical ventilation, poor living condition, and maternal education are ten risk factors of severe bronchiolitis caused by RSV.

## Figures and Tables

**Table 1:** The Modified Cincinnati Bronchiolitis Score (MCBS).

	0	1	2
Respiratory rate	Normal	Above tachypnea threshold (infant greater than 50 when not crying or agitated) accessory	

Muscles	Normal	Moderate retractions	Severe retractions

Air exchange	Normal	Localized decreased	Multi area decreased

Wheezes	None/end expiratory	Entire expiratory	Entire expiration and inhalation

*Evaluation*			
Mild: 0–2 points			
Moderate: 3-5 points			
Severe: 6-7 points			

**Table 2:** Epidemiological characteristics of children with acute bronchiolitis caused by RSV (*N* = 377).

Variables	Numbers of patients	Percentage (%)
*Sex*		
Boy	261	69.2
Girl	116	30.8
Area		
Urban	64	17.0
Suburb	271	71.9
Other	42	11.1
Age (month)		
≤6	217	57.5
7-11	84	22.3
12-17	52	13.8
18-24	24	6.4
Severity		
Mild-moderate	330	87.5
Severe	47	12.5

**Table 3:** The severity of acute bronchiolitis categorized by age.

Age (month)	Number of patients	Severe acute bronchiolitis, *n* (%)	Mild-moderate acute bronchiolitis, *n* (%)	*p* value
≤6	217	39 (18)	178 (82)	<0.01
6-11	84	6 (7.1)	78 (92.9)	
12-17	52	2 (3.8)	50 (96.2)	
18-24	24	0 (0)	24 (100)	

**Table 4:** The admission reasons of children with acute bronchiolitis caused by RSV (*N* = 377).

Admission reasons	Numbers of patients	Percentage (%)
Cough	18	4.8
Raspy cough	60	15.9
Wheezing	261	69.2
Cough and fever	27	7.2
Wheezing and fever	11	2.9

**Table 5:** The clinical characteristics of children with acute bronchiolitis caused by RSV (*N* = 377).

Symptoms and signs	Numbers of patients	Percentage (%)
Cough	377	100
Runny nose	377	100
Tachypnea	377	100
Labored breathing	374	99.2
Sibilant rales	373	98.9
Wheezing	370	98.1
Tachycardia	355	94.2
Fever	293	77.7
Sonorous rales	50	13.3
Fine crackles	130	34.5
Gray skin	3	0.8

**Table 6:** Chest X-ray of children with acute bronchiolitis caused by RSV (*N* = 377).

Chest X-ray images	Numbers of patients	Percentage (%)
Bronchiolitis	377	100
Air trapping lungs	124	32.9
Lungs without air trapping	253	67.1

**Table 7:** The risk factors of severe acute bronchiolitis caused by RSV (*n* = 377).

Risk factors		Numbers of patients	Severe acute bronchiolitis, *n* (%)	Mild-moderate acute bronchiolitis, *n* (%)	OR (95% CI)	*p* value
Sex	Male	261	35 (13.4)	226 (86.6)	1.34 (0.67-2.69)	>0.05
	Female	116	12 (10.3)	104 (89.7)		

Age (months)	≤6	217	39 (18)	178 (82)	4.16 (1.89-9.18)	<0.05
	>6	160	8 (5)	152 (95)		

Preterm birth	Yes	15	6 (40)	9 (60)	5.2 (1.76-15.4)	<0.05
	No	362	41 (11.3)	321 (88.7)		

Breastfeeding under 6 months	Yes	28	11 (39.3)	17 (60.7)	5.6 (2.4-12.9)	<0.05
	No	349	36 (10.3)	313 (89.7)		

History of exposure to cigarette smoking	Yes	6	3 (50)	3 (50)	7.4 (1.45-37.96)	<0.01
	No	371	44 (11.9)	327 (88.1)		

Low birth weight	Yes	13	8 (61.5)	5 (38.5)	13.3 (4.15-42.77)	<0.01
	No	364	39 (10.7)	325 (89.3)		

Having sibling(s) under five years old going to kindergarten	Yes	23	15 (65.2)	8 (34.8)	6.6 (2.57–17.01)	<0.05
	No	145	32 (22.1)	113 (77.9)		

History of undergoing cesarean section	Yes	22	6 (27.3)	16 (72.7)	2.8 (1.06-7.75)	<0.05
	No	355	41 (11.5)	314 (88.5)		

History of mechanical ventilation	Yes	7	4 (57.1)	3 (42.9)	10.14 (2.19-46.8)	<0.01
	No	370	43 (11.6)	327 (88.4)		

Poor living condition	Disadvantaged	13	7 (53.8)	6 (13)	9.4 (3.03-29.5)	<0.01
	No	364	40 (11)	324 (89)		

Maternal education	Graduated from high school & further education	14	6 (42.9)	8 (57.1)	5.89 (1.9-17.8)	<0.05
	Nongraduated from highschool	363	41 (11.3)	322 (88.7)		

## Data Availability

Data and materials used and/or analyzed during the current study are available from the corresponding author on reasonable request.

## Abbreviations

RSV: Respiratory syncytial virus.
